# Crisscross multilayering of cell sheets

**DOI:** 10.1093/pnasnexus/pgad034

**Published:** 2023-02-03

**Authors:** Trinish Sarkar, Victor Yashunsky, Louis Brézin, Carles Blanch Mercader, Thibault Aryaksama, Mathilde Lacroix, Thomas Risler, Jean-François Joanny, Pascal Silberzan

**Affiliations:** Laboratoire PhysicoChimie Curie UMR168, Institut Curie, Paris Sciences et Lettres, Centre National de la Recherche Scientifique, Sorbonne Université, 11 Rue Pierre et Marie Curie, 75248 Paris, France; Laboratoire PhysicoChimie Curie UMR168, Institut Curie, Paris Sciences et Lettres, Centre National de la Recherche Scientifique, Sorbonne Université, 11 Rue Pierre et Marie Curie, 75248 Paris, France; Department of Solar Energy and Environmental Physics, The Blaustein Institutes for Desert Research, Ben-Gurion University of the Negev, Midreshet Ben-Gurion, Negev, 84990, Israel; Laboratoire PhysicoChimie Curie UMR168, Institut Curie, Paris Sciences et Lettres, Centre National de la Recherche Scientifique, Sorbonne Université, 11 Rue Pierre et Marie Curie, 75248 Paris, France; Collège de France, Paris Sciences et Lettres, 11 place Marcelin Berthelot, 75231 Paris, France; Laboratoire PhysicoChimie Curie UMR168, Institut Curie, Paris Sciences et Lettres, Centre National de la Recherche Scientifique, Sorbonne Université, 11 Rue Pierre et Marie Curie, 75248 Paris, France; Laboratoire PhysicoChimie Curie UMR168, Institut Curie, Paris Sciences et Lettres, Centre National de la Recherche Scientifique, Sorbonne Université, 11 Rue Pierre et Marie Curie, 75248 Paris, France; Laboratoire PhysicoChimie Curie UMR168, Institut Curie, Paris Sciences et Lettres, Centre National de la Recherche Scientifique, Sorbonne Université, 11 Rue Pierre et Marie Curie, 75248 Paris, France; Laboratoire PhysicoChimie Curie UMR168, Institut Curie, Paris Sciences et Lettres, Centre National de la Recherche Scientifique, Sorbonne Université, 11 Rue Pierre et Marie Curie, 75248 Paris, France; Laboratoire PhysicoChimie Curie UMR168, Institut Curie, Paris Sciences et Lettres, Centre National de la Recherche Scientifique, Sorbonne Université, 11 Rue Pierre et Marie Curie, 75248 Paris, France; Collège de France, Paris Sciences et Lettres, 11 place Marcelin Berthelot, 75231 Paris, France; Laboratoire PhysicoChimie Curie UMR168, Institut Curie, Paris Sciences et Lettres, Centre National de la Recherche Scientifique, Sorbonne Université, 11 Rue Pierre et Marie Curie, 75248 Paris, France

**Keywords:** collective cell behaviors, active cell nematics, orientation, multilayers

## Abstract

Hydrostatic skeletons such as the Hydra's consist of two stacked layers of muscle cells perpendicularly oriented. In vivo, these bilayers first assemble, and then the muscle fibers of both layers develop and organize with this crisscross orientation. In the present work, we identify an alternative mechanism of crisscross bilayering of myoblasts in vitro, which results from the prior local organization of these active cells in the initial monolayer. The myoblast sheet can be described as a contractile active nematic in which, as expected, most of the +1/2 topological defects associated with this nematic order self-propel. However, as a result of the production of extracellular matrix (ECM) by the cells, a subpopulation of these comet-like defects does not show any self-propulsion. Perpendicular bilayering occurs at these stationary defects. Cells located at the head of these defects converge toward their core where they accumulate until they start migrating on top of the tail of the first layer, while the tail cells migrate in the opposite direction under the head. Since the cells keep their initial orientations, the two stacked layers end up perpendicularly oriented. This concerted process leading to a crisscross bilayering is mediated by the secretion of ECM.

**Significance Statement**

Hydrostatic skeletons such as the Hydra's consist of two stacked perpendicular layers of muscle cells that first assemble together and then organize with this crisscross orientation. The present article explores an alternative mechanism in vitro that relies on a prior organization of the cells in the monolayer. The initial monolayer can be described as an active liquid crystal phase in which, because of the production of ECM proteins by the cells, a fraction of the intrinsic topological defects remains practically stationary. Perpendicular bilayering occurs at these stationary defects where the monolayer eventually splits into two stacked perpendicular layers that migrate in antiparallel directions one on top of the other. This process highly depends on the cell-secreted ECM.

## Introduction

In contrast with epithelial cells that can be cultured in vitro as bidimensional monolayers over long periods of time, most other cell types grown on planar substrates expand in the third dimension after confluence. The ability to remain a monolayer can be related to contact inhibition of proliferation by which the division time increases with the cell density (1–5). Other regulation mechanisms, such as the extrusion and apoptosis of individual cells from the monolayer above a certain cell density, have also been invoked (5–7).

Non-epithelial cell types that do not retain a purely bidimensional organization after confluence develop multicellular 3D structures. They can take the form of aggregates on top of the initial monolayer (for instance neural progenitor cells [NPCs] in vitro (8)), or adopt a stratified structure in which the cells differentiate as the multilayered structure develops (for instance, skin epidermis or thymus (9)). Interestingly, transformed epithelial cells that express an oncogene also develop 3D structures after confluence, often coupled with the down-regulation of cell–cell adhesions in the context of the Epithelial-to-Mesenchymal Transition (1, 2, 10–13). Because of the physiological importance of this 2D-to-3D transition, strategies to include these 3D aspects have been developed when engineering functional tissues in vitro. These efforts have led to the development of Multilayered Cell Cultures and organs or tumors “on chip” (14–16).

Surprisingly, the mechanism by which mammalian cell monolayers expand into a multilayered structure remains elusive. This transition might be initiated by isolated cells in the monolayer that would extrude out of the layer and proliferate in such way as to develop a second layer progressively covering the surface of the first one. In an alternative, more collective, mechanism, the initial monolayer “splits” into two stacked layers that then expand by migration and/or proliferation while, possibly, differentiating to organize in a mature stack. Another salient related question relates to the locus of the initiation of multilayering: does it occur at random locations in the monolayer? Is it related to a particular cell or group of cells somehow different from the other cells? Or to the local organization of the cells in the initial monolayer?

It is worth noting here that the fate of a cell that has been extruded from a monolayer is both cell type-dependent and context-dependent. For instance, MDCK epithelial cells tend to enter apoptosis and die after having been apically extruded from the monolayer (6, 7), but they remain alive and incorporate into tridimensional rims when they are located next to a physical boundary (7). Another example of the impact of physical constraints is given by elongated cells that orient along a common direction and extrude preferentially at singularities in the supracellular organization of the cells (i.e. at topological defects) (8, 17).

Furthermore, it has been shown that the multilayered organization of spindle-shape fibroblast-like cells from chick embryos depends on the tissue the cells originate from. Indeed, the angle between successive layers varies from uncorrelated (cornea cells), to 0° (parallel alignment, skin cells) or 90° (perpendicular or “crisscross” alignment, heart cells) (18, 19). When contracted/relaxed independently, stacked perpendicular muscle layers allow for complex functions and exploration of the third dimension as exemplified by hydrostatic skeletons that actuate the hydra (20, 21) or the intestines (22), as well as by muscular hydrostats such as animal tongues (23). As a matter of fact, such principles are used in biomimetic designs of “soft robots” (24).

In the present article, we study the physical and biological processes responsible for the development of a cell monolayer into a crisscross bi- and eventually multilayer.

The recent theories of out-of-equilibrium active matter (25, 26) provide a general framework to model biological tissues, allowing to understand some aspects of the mechanical behavior of cell monolayers (27–31). In particular, populations of elongated cells have been successfully described as active nematic phases in which cells have no long-range positional order but share a common orientation over multiple cell lengths (31). These continuum theories have for instance provided a framework to understand the generation of spontaneous cell flows in confined monolayers of myoblasts or retinal cells (32), the critical role of the topological defects associated with such symmetries (33), or the emergence of low-Reynolds number turbulence in monolayers of bronchial epithelial cells (29).

In addition to these physical mechanisms, it has been shown that the multilayering process is directly related to the production of extracellular matrix (ECM) proteins such as collagen or fibronectin, by the cells themselves (34–37). Therefore, it is expected that physical and biochemical contributions concur to the development of a crisscross bilayer.

More specifically, we study here how spindle-shaped C2C12 murine myoblasts transit locally from a bidimensional active nematic monolayer to a crisscross multilayer. After confluence, most of the classical +1/2 defects that are characteristic of a contractile active nematic are mobile. Over time, they pairwise annihilate with −1/2 defects. However, a fraction of these defects remains stationary. We find that bilayers nucleate at these stationary +1/2 defects. The peculiar asymmetric velocity field around one of these defects results in an accumulation of cells at the head of the defect. The defect then evolves into a bilayer in which the cells originally at the head of the defect migrate collectively *on top* of the initial monolayer while the cells originally pertaining to the tail migrate *underneath* the head cells in an ECM-dependent process. Since they keep their initial orientations, the cells of the two stacked layers arrange in a crisscross structure. We show that the onset of the multilayering process is largely controlled by the physics of this contractile active nematic system, while the organization and dynamics of the two stacked layers rely also on the ECM secreted by the cells.

## Results

**Fig. 1.**
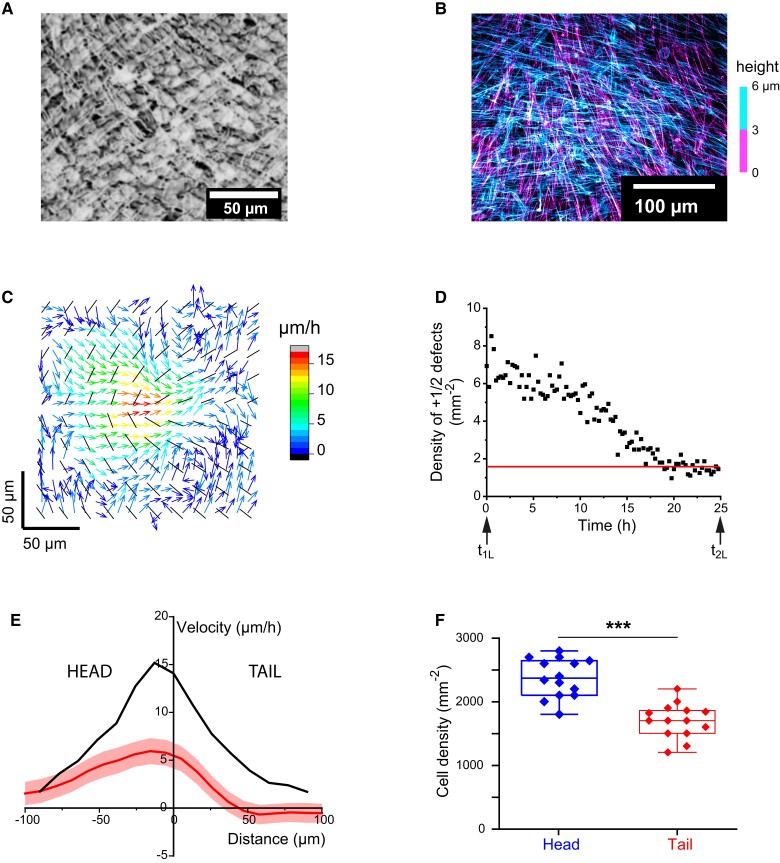
(A) C2C12 bilayer (hematoxylin stained fixed cells, bright field, contrast-enhanced) showing the perpendicular orientation of the two stacked layers. (B) Confocal image of the same showing the two stacked layers at orthogonal orientations (Fixed cells, actin labeling. The color codes for the height.) (C) Before bilayering, the average orientation field (black lines) and the velocity field (colored arrows) measured at a +1/2 defect are consistent with a contractile active nematic description (average over 5,398 observations from 350 independent defects [three independent experiments]). (D) The surface density of +1/2 defects decreases with time but plateaus at typically 2 defects/mm² several hours before the onset of bilayering (representative experiment, three replicates). (E) Average velocity profiles along the axis of the +1/2 defects. Black line: all defects (N = 350), red line: immobile defects (N = 15, 3 independent experiments). x = 0 is the position of the core. Note the zero velocity in the tail of the stationary defects while the velocity in the head remains finite. Colored areas are the SEMs. (F) As cells of the head of the immobile defects flow toward their core, the cell density increases in the head. Measurements performed in the hour preceding bilayering (N = 15, 3 independent experiments).

As muscle cells organize frequently in perpendicular stacked layers in vivo (22), we chose to study the bilayering of C2C12 myoblasts. Sparse C2C12 cells plated on Fibronectin-coated glass proliferated, reached confluence (defining a time t_1L_) and then multilayered, reaching thicknesses up to 30 µm, 5 days after confluence (Figure S1). As hypothesized, these cells form striking crisscross bilayers (Fig. 1A, B). Being interested in the process by which a cell monolayer develops into two stacked perpendicular cell layers, we monitored the behavior of the cells after confluence and until the formation of a crisscross bilayer. We call the time of initiation of bilayering t_2L_. In the present work, we dissect the first steps of the formation of a crisscross bilayer in relation with both the local organization of the cells and the production of ECM proteins by the cells themselves.

In the following, we call “layer 1” the first cell layer directly in contact with the coverslip; subsequent layers are called “layer 2”, …, “layer n”.

Note that C2C12 cells are undifferentiated myoblasts that would eventually differentiate into myotubes if cultured in the right conditions of serum and growth factors (38). However, in the present experiments, we use cells that have experienced enough passages after confluence (up to 40) to be selected against differentiability (39, 40). Doing so, we can study the multilayering phenomenon with cells whose phenotype remains unchanged for the duration of the experiments.

### C2C12 cells organize in an active nematic monolayer

After reaching confluence, C2C12 cells self-organized in a bidimensional active nematic phase in which they oriented along a common direction and formed supracellular domains (31), as previously reported (8, 32, 33). This phase is characterized by typical 2D nematic topological defects (of topological charges −1/2 and +1/2) that position themselves between the domains of uniform orientation (Figure S2). Such defects can be readily identified in phase contrast or fluorescence images of the actin cytoskeleton, and the contractile or extensile nature of the system can be determined from the analysis of the cell flow around +1/2 defects (31). We first analyzed these flows during the first 10 hours following confluence. Taking advantage of the characteristic comet-like shape of the +1/2 topological defects, we superimposed a large number (5,398 observations of 350 different defects in three independent experiments) of them to access their average orientation field and the associated average velocity field (41) (Fig. 1C, Figure S3A, C). In the present situation, two counter-rotating vortices developed at +1/2 defects. The signs of the vorticities were characteristic of a contractile nematic system with flows at the defect's core directed toward the tail (29, 42, 43). Most of the +1/2 defects were therefore motile, heading “tail first” (Figure S4B).

### Stationary +1/2 defects

Since there was no creation of new defects after confluence, annihilation events involving pairs of defects of opposite charges resulted in a decrease of the surface density of +1/2 defects with time, as previously reported (27) (Fig. 1D). However, the defect density did not vanish but plateaued at 1–4 defects/mm², ∼ 5 h before t_2L_ (three replicates). Strikingly, the remaining defects were stationary within the monolayer (Figure S4A). Tracking them backwards in time showed that they actually kept their positions or moved very slowly down to 15 h before t_2L_ (see the 5h-trajectories in Figure S4A). This behavior was not caused by a global jamming of the monolayer (44), as individual cells still moved along the lines of the orientation field (Movie S1). More quantitatively, the speed of these defects remained smaller than 10 µm/h, whereas defects that disappeared before t_2L_ by annihilation moved at speeds of the order of 20 µm/h (Figure S4C). In the following, we refer to the defects that survive up to t_2L_ as stationary defects, and those that disappear before t_2L_ by annihilation as motile defects.

Cells located at the head of a stationary defect converged toward its core. In contrast, no significant flow was detected in the tail region (Figure S3B, D). As a result, the average velocity profile along the axis of an average +1/2 stationary defect was very asymmetric about the core with non-zero values only at the head (Fig. 1E). This observation is qualitatively reminiscent of the asymmetric flow field around defects in monolayers of NPCs (8) although the NPC system is extensile such that the flow is directed from the defect's tail toward its core and decreases sharply at its head. In the present contractile case, the flow distribution is opposite, with measurable flows only at the head of the defect. In addition, we observed an accumulation of C2C12 cells at the head of the stationary +1/2 defects (Fig. 1F). This cell accumulation built up over time and reached its maximum just before the formation of 3D structures. Analogy with ref (8) then strongly suggested that this 2D cell accumulation participated to the formation of 3D structures.

**Fig. 2.**
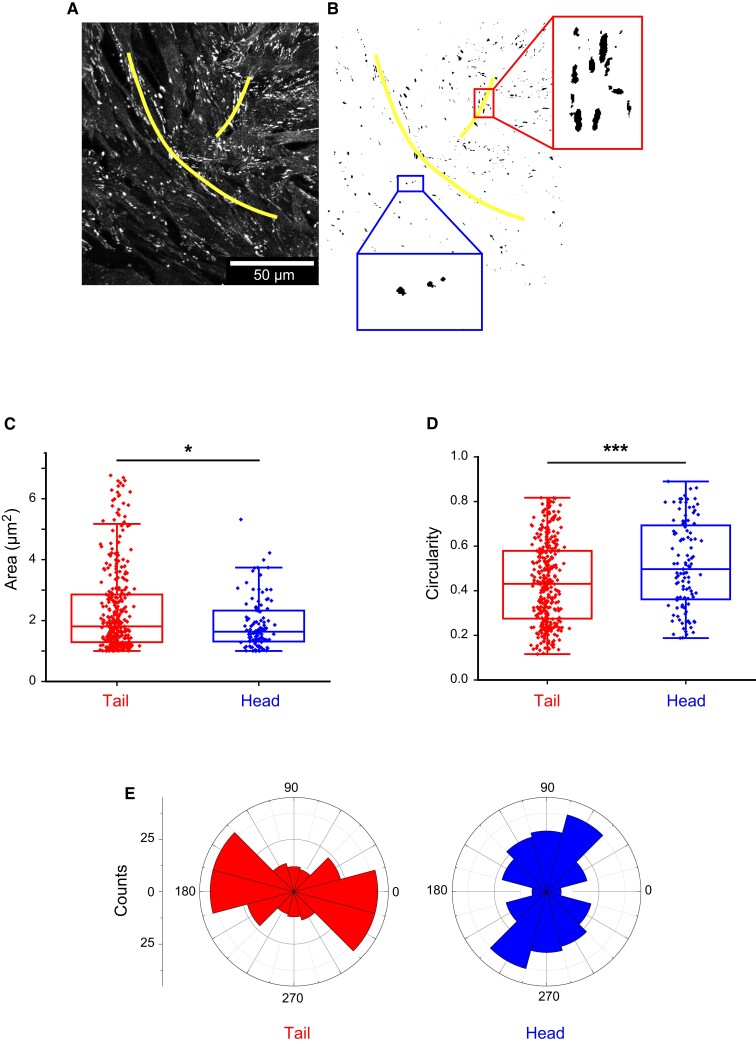
(A) Focal adhesions (FAs) (fixed cells, paxillin labeling) at a stationary +1/2 defect just before bilayering. The defect has been outlined in yellow. (B) Thresholded image. Note the elongated FAs in the tail while they are more circular in the head. (C, D) Quantification of the area and circularity of the FAs in the tail and head regions of the stationary defects (4 independent defects from two independent experiments). Although the distributions of the FAs’ areas are very similar, FAs in the tail are more elongated (circularity in the head = 0.4 ± 0.2 (SD); in the tail = 0.5 ± 0.2 (SD); p = 5E-5). (E) Corresponding histograms of the orientations of the FAs in the tail (red) and in the head (blue) of stationary defects. The defects are oriented along the 0° direction.

To access the force field at these stationary +1/2 defects, we took advantage of the sensitivity of Focal Adhesions (FAs) to an external force (45, 46). FAs were imaged via the labeling of paxillin. Close to the core of the defects, they were classically positioned at the end of stress fibers (Fig. 2A, Fig. 4D). Although their areas (Fig. 2C) and their surface densities were similar in the head and in the tail, the cells of the tail developed slightly more elongated FAs (Fig. 2D) oriented in the tail direction (Fig. 2E). This anisotropy of the FAs may result from a directed force acting on the tail cells.

### Onset of bilayering at stationary +1/2 defects

Since +1/2 topological defects have been previously shown to play an important role in apical cell extrusion processes (8, 17), we now question how the transition of C2C12 cells from a monolayer to a bilayer can be related to this particular cell organization and to the associated velocity and force fields.

Previous reports have emphasized the importance of +1/2 defects in the formation of 3D disorganized clusters from a monolayer (8). However, in contrast with these previous studies, the transition evidenced here is characterized by a collective bilayering process and not by the extrusion of a disorganized cell aggregate.

**Fig. 3.**
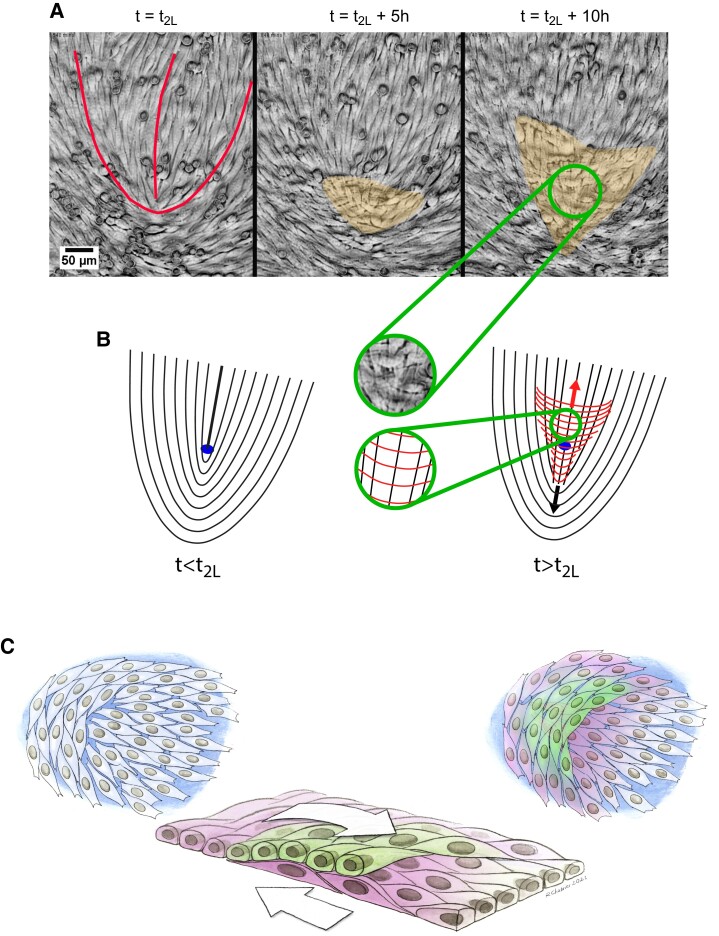
(A) Sequence of snapshots (phase contrast) illustrating the progression of the bilayering. The orange area highlights the crisscross bilayer. The outline of the defect at t = t_2L_ is shown as a red line. See full movie in Movie S2. (B) Schematic of the bilayering process. The red lines correspond to the orientation field lines of layer 2 while the black lines refer to layer 1. The blue dot shows the initial position of the core. Note the crisscross organization of the two layers when t > t_2L_ (insets). (C) Artist view of the bilayering process. Note that this is an idealized picture. In reality, layers 1 and 2 are very thin and their heights are not as well defined as depicted. As a consequence, layers 1 and 2 are somewhat intertwined. Moreover, parallel bilayering, which results from single cells being extruded from layer 1, is not represented in this panel.

In our experiments, the motile self-propelled defects annihilated with −1/2 defects and disappeared from the monolayer before t_2L_. The 2D-3D transition occurred at the stationary +1/2 defects via the splitting of the monolayer into two stacked layers. More precisely, out of 15 identified stationary “final” defects (from three independent experiments), 14 gave rise to a crisscross bilayer. Further evolution of the system was driven by the migration of these two layers. Indeed, we observed that cells initially at the head of the defect but close to its core, collectively migrated *on top* of the cells of the first layer pertaining to the tail of the initial defect. In parallel, the cells initially pertaining to the tail of the defect moved collectively *underneath* the cells originally located at the head of the defect (Fig. 3, Movies S2, S3).

It is worth noticing here that, more than 50 years ago, by carefully observing sheets of embryonic lung fibroblasts, T. Elsdale and co-workers concluded that crisscross bilayering originated at immobile frontiers between well-oriented domains (34). Given the technology of the time, this was a truly remarkable conclusion. Here, we largely confirm this part of Elsdale's analysis. Moreover, we show that these multilayers form at the stationary topological +1/2 defects localized at these frontiers.

### Organization and dynamics of the bilayer

At the onset of bilayering at a stationary +1/2 defect in layer 1, the cells of layer 2 originating from the head of the defect migrated on top of layer 1. In parallel, cells of layer 1 originating from the tail of the defect crawled under layer 2. During this process, cells of both layers kept their initial orientations. Therefore, the cells of layer 1 and those of layer 2 were oriented along perpendicular directions with respect to each other in the region of overlap (Movies S2 and S3; Figure S5). This perpendicular alignment corresponds to previous observations of fibroblast-like cells from lung (47) or heart (18). Interestingly, disrupting the cadherin-based cell–cell adhesions by addition of EGTA did not abrogate perpendicular bilayering (three replicates) (Figure S6).

Perpendicular bilayering was not the only way by which the thickness of the cell sheet increased: In defect-free regions, cells dividing in the high-density confluent monolayer could not intercalate in the monolayer and adhere to the substrate due to lack of space. They partially incorporated in layer 1 while keeping their initial orientation and contributed to the progressive development of layer 2. Therefore, this configuration yielded a gradual increase of thickness, in which cells pertaining to both layers were all oriented along the same direction. We call this process “parallel bilayering”. We emphasize that, whereas perpendicular bilayering is clearly a collective event implying the concerted action of a group of cells, parallel bilayering is an individual insertion event originating from cell division. Although both mechanisms contribute to the multilayering of the cell system, they are clearly independent. Yet, perpendicular and parallel bilayering occurred simultaneously, which, together with the small and uneven thickness of the cell layers, contributed practically to limit the z-resolution in confocal stacks.

After completion of layer 2, the two layers continued to evolve until large parallel domains coexisted with crisscross domains. The mechanisms of reorientation ruling this maturation of the bilayer are different from the ones studied here and are beyond the scope of the present paper that focuses on the onset of crisscross bilayering.

We now turn to the dynamics of the perpendicular bilayering. The migration modes of layers 1 and 2 were primarily collective with only rare events of cells detaching from their neighbors at the respective front edges. Along the axis of the initial defect, the front edge velocities of layers 1 and 2 with respect to the glass substrate measured by cell tracking were comparable (respectively, 12 ± 4 µm/h (n = 7) and 15 ± 7 µm/h (n = 5)). Recirculating flows were observed on the sides (Movie S1).

Importantly, the direction of collective migration was parallel to the cells’ main axis for layer 1 but orthogonal to it for the cells of layer 2 (Movies S1 and S2, S3). Note that isolated C2C12 cells migrate along of their long axis.

We conclude that perpendicular bilayering initiates at +1/2 stationary defects because of the accumulation of cells at the defect head. The monolayer then splits into two stacked layers that migrate actively in antiparallel directions, one on top of the other. Because they keep their initial orientations in this process, the cells of the two layers end up perpendicularly oriented. We also observed that the same mechanism leads to the formation of crisscross bilayers of Retinal Pigment Epithelial (RPE1) cells at similarly stationary +1/2 defects (Figure S7) after annihilation of all self-propelled +1/2 defects. Note that RPE1 cells, as C2C12 cells, organize in a nematic contractile active monolayer (32).

### Extracellular matrix secretion mediates multilayering

The migration of a second layer on top of the first one questions the biochemical nature of the surface on which the cells of layer 2 collectively migrate on top of layer 1. In particular, extrusion of NPC or MDCK cells did not lead to a well-developed second layer but to cell mounds (8) or apoptotic cells (17).

Here, we hypothesized that interactions between successive layers were mediated by cell-secreted ECM proteins, and we therefore proceeded to precisely localize some of its prototypic components in the stacked layers.

We focused on three major proteins of the ECM (48): (i)collagen IV, which is the structural element of ECM, (ii)fibronectin, and (iii)laminin, that both directly interact with integrins (36, 49). In addition, information on cell anchoring in these multilayered systems was also obtained by imaging paxillin that is part of the Focal Adhesion (FA) complex (50, 51).

**Fig. 4.**
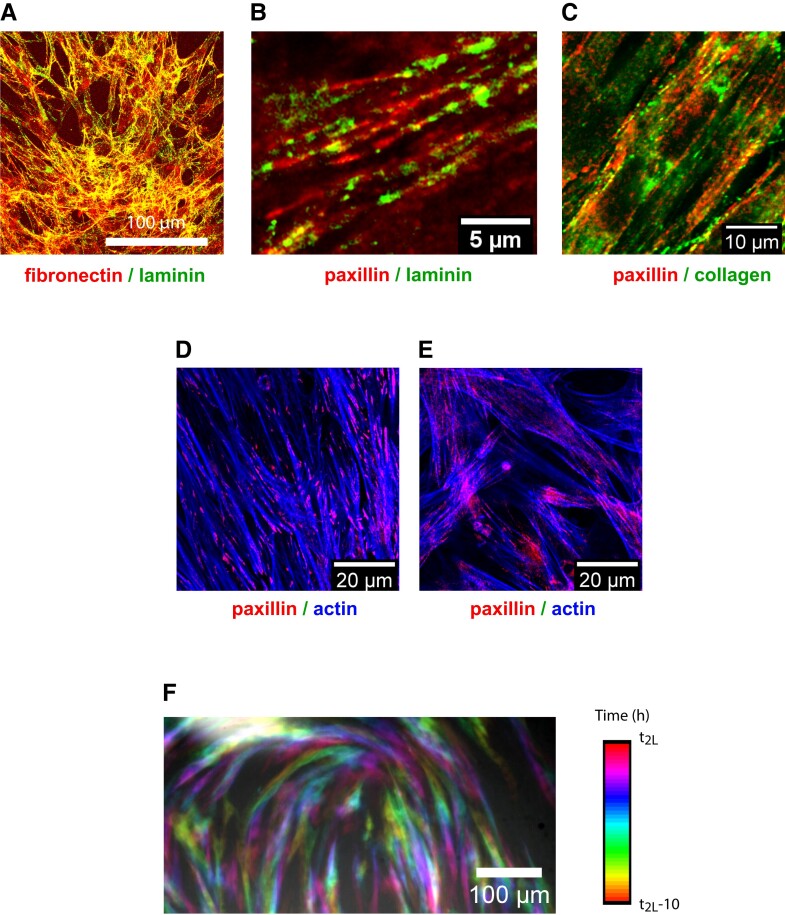
(A) Fibrillar ExtraCellular Matrix mediates cell–cell and cell–substrate contacts (image acquired in layer 2 in a well-developed multilayer). Maximum intensity projection of a confocal stack. Fixed cells. The mostly yellow color demonstrates the colocalization of laminin and fibronectin. (B, C) ECM (laminin (B) and collagen (C)) localizes between the cells of layers 1 and 2 in a multilayered sample (these images are confocal slices acquired at the interface between layer 1 and layer 2 in a well-developed multilayer). Note the colocalization of ECM proteins with the Focal Adhesions (FAs) (labeled by paxillin), characteristic of the 3D organization of the cells. (D, E) The FAs structure is very different for layer 1 interacting with glass (D) compared with the subsequent layers (E). For cells adhering on glass, one recovers the classical well-defined FAs at the extremities of the stress fibers (D). In the case of multilayers, cell–cell adhesion is mediated by the cell-secreted ECM. The fibrillar structure of the FAs that colocalizes with actin within the multilayered assembly is then typical of a tridimensional organization of the cells (E) (confocal slice at the basal side of layer 1 (D) and in the multilayer, 10 µm above (E) Fixed cells). (F) Superimposed positions of the cells in a stationary defect during a 10 h sequence (starting at t_2L_—10 h and ending at t_2L_). For this analysis, 10% of the cells were Actin-mCherry so that they could be tracked independently (see Movie S1). All images of the movie have been colored according to time and summed up. The cells move actively along the orientation lines of the defect explaining how the defect can be stationary and the cells motile.

Cells were fixed at different stages of multilayering and the proteins of interest were imaged by confocal microscopy. On well-formed multilayers, we systematically observed the presence of large amounts ECM between cells (Fig. 4A, Figure S8). Paxillin imaging showed the presence of FAs for cells of all layers (Fig. 4B, C), confirming that cells of superimposed layers interacted with ECM via integrin-mediated adhesions. Therefore, it appears that cells secrete the ECM molecules that are necessary to ensure good adhesion between the successive stacked cell layers.

FAs looked similar for all layers except for layer 1, which was in direct contact with the Fibronectin-coated glass, while the other layers interacted with the ECM between their basal side and the apical side of their underlying cell layer (Fig. 4D, E). Except very close to the defect core at the mono- to bilayer transition, the adhesion sites of layer 1 on glass were well-defined FAs at the extremities of stress fibers as it is usually observed for cells spreading on ECM-coated glass (Fig. 4D). In the subsequent layers, the adhesions that the cells developed with their underlying cell layer were more fibrillar (Fig. 4E) and colocalized with the ECM with a pattern typical of 3D organization (50, 52). The adhesions of the cells of layer 1 with the glass substrate were therefore different from the ones developed between the stacked layers.

Adding low-concentration collagenase, which selectively degrades collagen, just before t_2L_ totally prevented the development of layer 2 (three replicates). The cells remained in the form of a monolayer for at least 10 h after the addition of the drug. The enzyme also destroyed the limited patches of layer 2 that had already begun to form (Movie S4). The relative low impact of collagenase on the adhesion of layer 1 to glass confirmed our previous observations that FAs of layer 1 behave differently from those of layer 2 and subsequent ones.

Interestingly, the secreted ECM fibrils were oriented in the direction of the cells that secreted them, i.e. in the direction of the cells of the first layer for the formation of the second layer (Fig. 4A-C). Since cells of the head of the comet migrate on top of layer 1 in the direction given by the cell bodies of this first layer (i.e. the direction of the tail), this collective motion may be mediated by the ECM secreted by these cells. During this process, the layer 2 migrating cells did not reorient and therefore migrated perpendicularly to their long axis. Layer 1 migrated actively underneath layer 2 along the direction of its constituent cells (therefore perpendicular to the cell orientation of layer 2).

## Discussion

Cultures of C2C12 cells shortly after confluence show the characteristic flow field of an active nematic contractile system around +1/2 defects. However, a fraction of these defects remains stationary in the 15 h preceding bilayering. These stationary defects eventually give rise to perpendicular bilayering events.

In the case of extensile systems, a skewed velocity distribution has previously been attributed to an “anisotropic friction” that takes larger values when the velocity of the cells is perpendicular to their orientation (8, 53). For extensile systems, this velocity profile results in an accumulation of cells that eventually extrude from the monolayer.

In the present case, at high cell density, some of the defects of layer 1 drastically slow down. We can think of several explanations for this phenomenon: Recent work has estimated the localized force acting on the core of a +1/2 defect necessary to stop its motion (43). It was found that this stall force decreases as a power law of the size of the defect core and could for this reason be as small as the force that can be exerted by FA complexes, explaining that some defects can then remain stationary. Yet, the velocity pattern around a defect stalled by a local force acting on its core is not recapitulated by the experimental data. Another possibility would be that the stalling of these defects is mediated by a disordered lattice of obstacles that would freeze their positions in the otherwise dynamical monolayer (54, 55). In the present case, C2C12 myoblasts have been previously shown to secrete large quantities of ECM (37). Heterogeneities in the basal deposition of ECM by the cells on the glass surface (56) could act as a self-generated such lattice of obstacles. Finally, we could imagine that, as the cells move on the glass coverslip, they remodel the ECM coating, in such a way that their followers tend to migrate along the same path. This would constitute a history-dependent freeze of the defect configuration, such as that envisioned in (57). The difficulty is then to picture how the head cells eventually migrate on top of the cells of the first layer, leaving the track they were previously migrating along.

Our observations show that the laminin layer secreted by the cells on the glass surface follows the local orientation of the cells of layer 1 (Figure S9A). Moreover, we observe that the trajectories of the cells next the defect core follow the lines of the orientation field (Fig. 4F), which would be compatible with a remodeling of the ECM by the cells. This process would result in guiding “tracks” shaped as the defect itself, which would in turn direct the cells by contact guidance. We note however that the orientation of other components of the ECM such as collagen (Figure S9B), is not as marked as the laminin's.

In any case, compared to the expected self-propelled defects in an active phase, the cell flows at stationary defects are considerably perturbed. Along the axis of the defect, the only subsisting displacements are at the head, directed toward the core and are perpendicular to the cells’ long axis. The elongated shape of the FAs of the tail cells suggests an oriented force dipole resulting from the high cell density in the head (45).

When the cell density is too large, the system does not remain bidimensional. The head cells are expelled from the first layer and cross over to form a layer 2 migrating on the top of layer 1, while layer 1 migrates under the head cells in the opposite direction.

ECM secretion is critical to enable the migration of layer 2 on top of layer 1 as demonstrated by the inhibition of the formation of a bilayer in the presence of collagenase. Indeed, MDCK epithelial cells that only secrete these proteins at their basal side (58–60) do not provide the adequate template for the spreading of another layer on the top of the first one and therefore do not form bilayers. Rather than multilayering, MDCK cells individually extrude at the defect sites but cannot adhere to the apical side of the monolayer and undergo apoptosis (17, 61, 62). Since disrupting N-cadherin-mediated C2C12 cell–cell contacts with EGTA did not abolish the bilayering process, the role of cadherins is secondary compared to that of the cell–ECM interactions that appear to mediate all cell–cell contacts (37).

Interestingly, cells migrating on top of layer 1 to form layer 2 keep their initial orientation, meaning that their velocity is perpendicular to their long axis. This mode of “sideways” migration in collective migration, although uncommon, has occasionally been observed for instance at the front edge of a migrating monolayer of Zebra Fish epicardial cells (63) or at the edge of closing wounds in fibroblasts’ monolayers (64). Since some features of the cells such as the morphology of their FAs bears some characteristics of a 3D environment, they may also have switched to a mode of migration intermediate between those of 2D and 3D.

The speed of the collective migration after the initiation of the bilayering is in the range ∼12–15 µm/h for both cell layers (in both cases relative to the glass substrate). The speed of layer 1 is within the range of values classically measured in wound-healing experiments. However, cells forming layer 2 collectively migrate on a counter-migrating substrate (the layer 1). They run the wrong way on a treadmill at a large relative speed of ca. 25–30 µm/h. These cells keep the orientation of the first layer and therefore nucleate a second layer in which the cell bodies are oriented perpendicularly to its direction of motion.

At the end of this process, the structure adopted by the cells is a perpendicular crisscross orientation of the cells of layers 1 and 2. Since other cell types, such as RPE1 cells, bilayer with a similar mechanism, it seems safe to assume that this mechanism may generically be at play in nematic cell sheets. We also note that, although +1/2 defects control the crisscross bilayering in a boundary-free C2C12 monolayer, integer defects, either asters or spirals, are at play when the same cell type is confined in small domains. This configuration gives rise to other complex multilayer organizations (65).

Altogether, this study highlights the complex interplay between physical and biochemical cues resulting in the crisscross organization of stacked layers of muscle cells. This mechanism is qualitatively distinct from the one at play in the development of crisscross muscle layers in hydrostat-like structures such as the small intestine (22) or the developing hydra, where the endoderm and the ectoderm have perpendicular orientations (20, 21). Indeed, in both cases, the bilayer first forms and the muscle fibers then orient themselves to achieve the crisscross organization. In contrast, the present paper describes a mechanism in which the cells keep a memory of their orientation in the monolayer at +1/2 defects, while they form the bilayer. Here, bilayering and crisscross orientation appear simultaneously. Because of the large number of tissues in vivo in which a nematic organization of the cells has recently been identified, and the now well-recognized potential impact of topological defects on 2D-to-3D transitions in these cell assemblies, we speculate that there may exist in vivo situations where the general mechanism identified in the present study is at play.

## Methods

### Cell culture and plating

The experiments were conducted with (i) wild type C2C12 cells (gift from Clotilde Thery [Exosomes and Tumor Growth, INSERMU932, Institut Curie]), and (ii) CRISPR labeled Actin mCherry cells.

Some experiments were conducted with RPE1 cells (33) cultured in the same conditions as C2C12 cells.

Cells were cultured in standard DMEM medium (ThermoFisher scientific) supplemented with 10% FBS (Gibco) and 1% Pen-Strep Penicillin–Streptomycin mixture (Gibco). Experiments involving fluorescence microscopy of any kind were performed with phenol red-free Gibco DMEM, high glucose, HEPES, supplemented with 10% FBS (Gibco), 1% Penicillin–Streptomycin (Pen-Strep) mixture (Gibco), and Glutamax supplement.

For all experiments, cells were plated on Fibronectin-coated (10 µg/mL) glass coverslips. Note that, due to their large passage number after reaching confluence (more than 30), the cells have lost their ability to differentiate.

The concentration of EGTA (Sigma) used for calcium chelation to disrupt N-cadherins was 2.5 mM. The concentration of collagenase (Sigma) used was 2.5 microunits/mL.

### Microscopy

The phase-contrast and epifluorescence time-lapse movies on live cells were acquired with an automated Olympus X71 microscope, equipped with 4x, 10x, and 20x objectives while maintaining 37°C, 5% CO_2_ partial pressure and 95% relative humidity (Life Science incubator).

The acquisitions were performed with a CCD camera (Retiga 4000R, QImaging) controlled by Metamorph (Universal Imaging). The typical frequency of image acquisitions was 15 minutes.

For confocal time-lapse imaging, we used an inverted laser scanning confocal microscope with spectral detection (LSM700—Zeiss) equipped with a CO_2_ incubator to observe fluorescent live cells (SiR-Actin labeling) with a 25x oil objective. The typical duration between acquisitions was 30 minutes.

For super-resolution confocal imaging on fixed cells (Figs. 2 and 4; Figs. S1, 9), we used an inverted Laser Scanning Confocal Microscope with Spectral Detection and Multi-photon Laser (LSM880NLO/MaiTai Laser—Zeiss/Spectra Physics), 63x oil immersion objective combined with super-resolution technique Airyscan.

Tracking experiments were performed by using mixtures with 10% Crispr-labeled Actin mCherry cells. Observations were performed in epifluorescence. Thanks to the relatively large depth of field, cells of both layers could be imaged simultaneously and then tracked manually.

### Cell fixation and fluorescent staining

To fix the cells, a 16 minutes of 4% (wt/vol) PFA treatment was followed by permeabilization by 0.1% Tween-20 (10 minutes). Fibronectin staining was an exception, for which ice-cold 100% methanol was used for fixation (no permeabilization step). DAPI was used for the nuclei, phalloidin-Tritc and SiR-actin dye were used for actin, and respective primary and secondary antibodies were used for respective proteins (see Table S1 for details).

In Fig. 1A, the cells were fixed in ice cold ethanol and stained with acidic Harris Hematoxylin solution (HHS16, Sigma) (34).

SiR-actin and SiR-DNA (Cytoskeleton Inc) were also used for live cell imaging according to the manufacturer protocols (Final concentration 1 µM).

### Analysis of the images

Analysis of the images was performed using FIJI (66) or Matlab (MatWorks).

FAs (Fig. 2) were analyzed via paxillin imaging. The images were thresholded at the same level after equalization of the histograms. Measurements were then performed with Fiji on FAs larger than 1 µm² and smaller than 7 µm^2^ (4 FOVs, 2 independent experiments). The circularity was defined as $4\pi \cdot \frac{area}{\;perimeter^{2}}$.

The density of cells (Fig. 1D) was mapped by manually counting the nuclei (SiR-DNA labeled cells).

### Orientation and velocity fields mapping

The orientation field was acquired by computing the local gradient structure tensor with the FIJI “OrientationJ” plugin, as described in (27, 67). To map the velocity fields, Particle Image Velocimetry (PIV) (41) was performed with the Matlab toolbox PIVlab 1.41 (68) with 16 pixels-windows, which corresponds to 29.7 µm, and 50% overlap. The time intervals between successive frames were 15 minutes or 30 minutes depending on the experiment.

### Defect detection and analysis

Defects were detected by mapping a local order parameter in windows of size Ω = 27.8 µm * 27.8 µm:


$$Q_{local}(\Omega )=\sqrt{\langle cos2\Theta \rangle_{(x,y)\in \Omega }^{2}+\langle sin2\Theta \rangle_{(x,y)\in \Omega }^{2}}$$

whose minima correspond to the position of the defects. Their charge and orientation was measured by calculating the variations of angle along a virtual loop around the core (29).

The extracted orientation and velocity fields at +1/2 defects were then processed to average these two fields.

## Supplementary Material

pgad034_Supplementary_Data

## Data Availability

The data (images and videos) that support the findings of this study and that are not in the Supplementary material file, have been deposited in Zenodo at https://doi.org/10.5281/zenodo.7547084
