# Supplementary material for: Crisscross multilayering of cell sheets
Source: PNAS Nexus. 2023 Feb 3;2(3):pgad034. doi: 10.1093/pnasnexus/pgad034 (PMC10019763; doi:10.1093/pnasnexus/pgad034)
Supplement: pgad034_Supplementary_Data [file pgad034_supplementary_data.zip › supp figures movies table with captions nexus EGTA.PDF]

Sarkar et al. « Crisscross Multilayering of cell sheets »

Supplementary Material

- Figures S1 to S9
- Table S1
- Captions of Movies S1 to S4

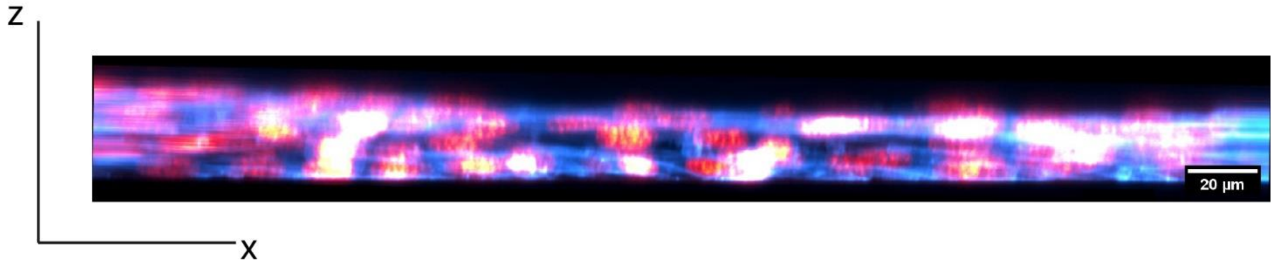


**<u>Figure S1:</u>** Confocal xz section of a fixed mature multilayered cell sheet, t=5 days (actin blue, nuclei red). The stack consists of ~4 cell sheets on top of each other. Its thickness goes up to 30 microns.

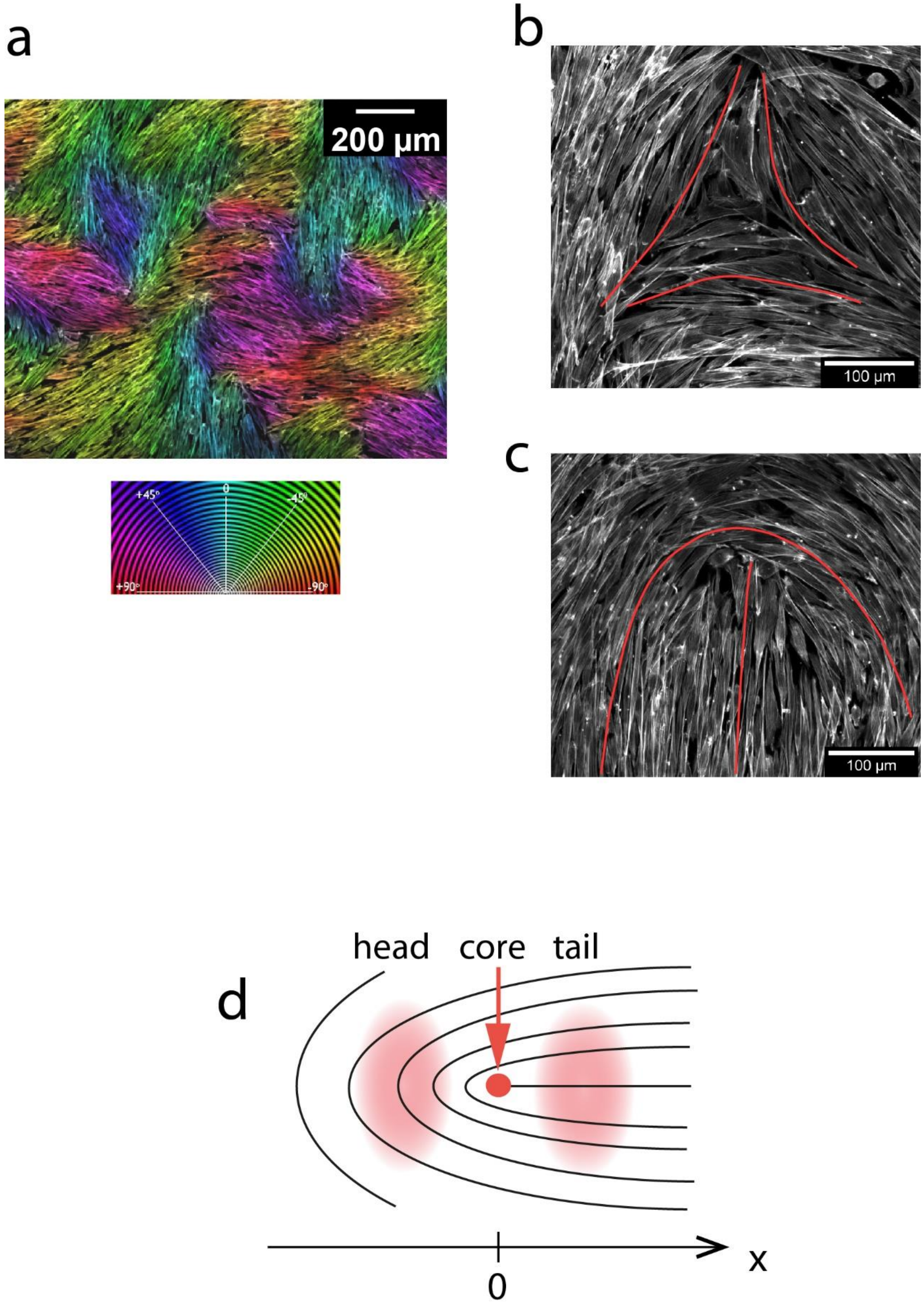


**Figure S2**: a/ C2C12 cells at confluence self-organize in well-aligned domains between which topological defects position themselves. The color codes for the orientation. b/ -1/2 defect (actin labeling), c/ +1/2 defect (actin labeling). Red lines outline the defects. d/ Schematic of a +1/2 comet-like defect and notations used in the article.

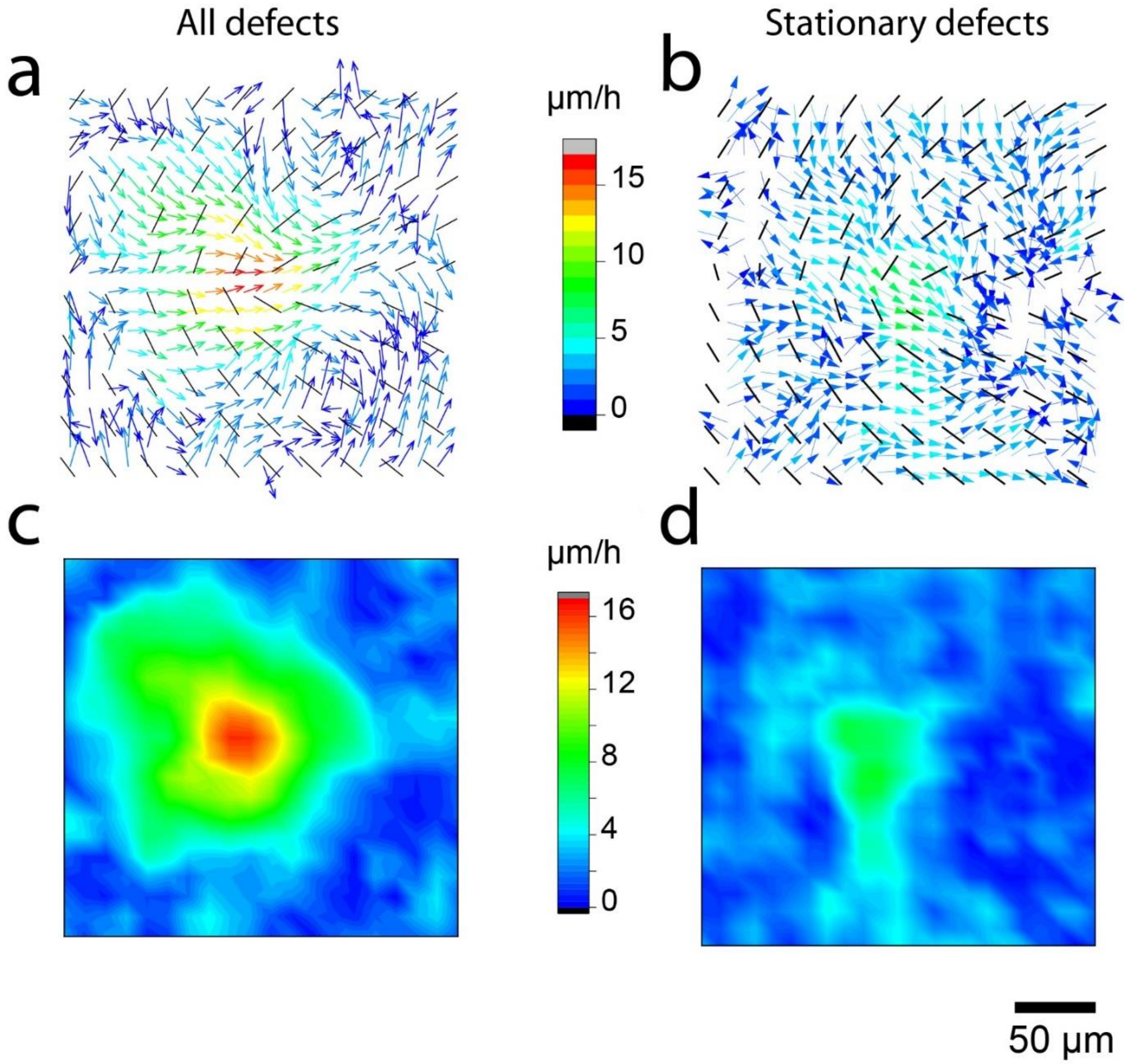


**Figure S3:** Velocity fields for the entire population of defects (a, c) and the subpopulation giving rise to bilayers (b, d). Velocities are visualized with colored arrows (a,b). The color codes for the velocity amplitude. Orientations of the cells are given by the black lines (a,b). c) and d) show the amplitude of the velocity. All defects: 5398 observations from 350 defects in 3 independent experiments. Defects giving rise to bilayers: 300 observations from 15 defects in 3 independent experiments.

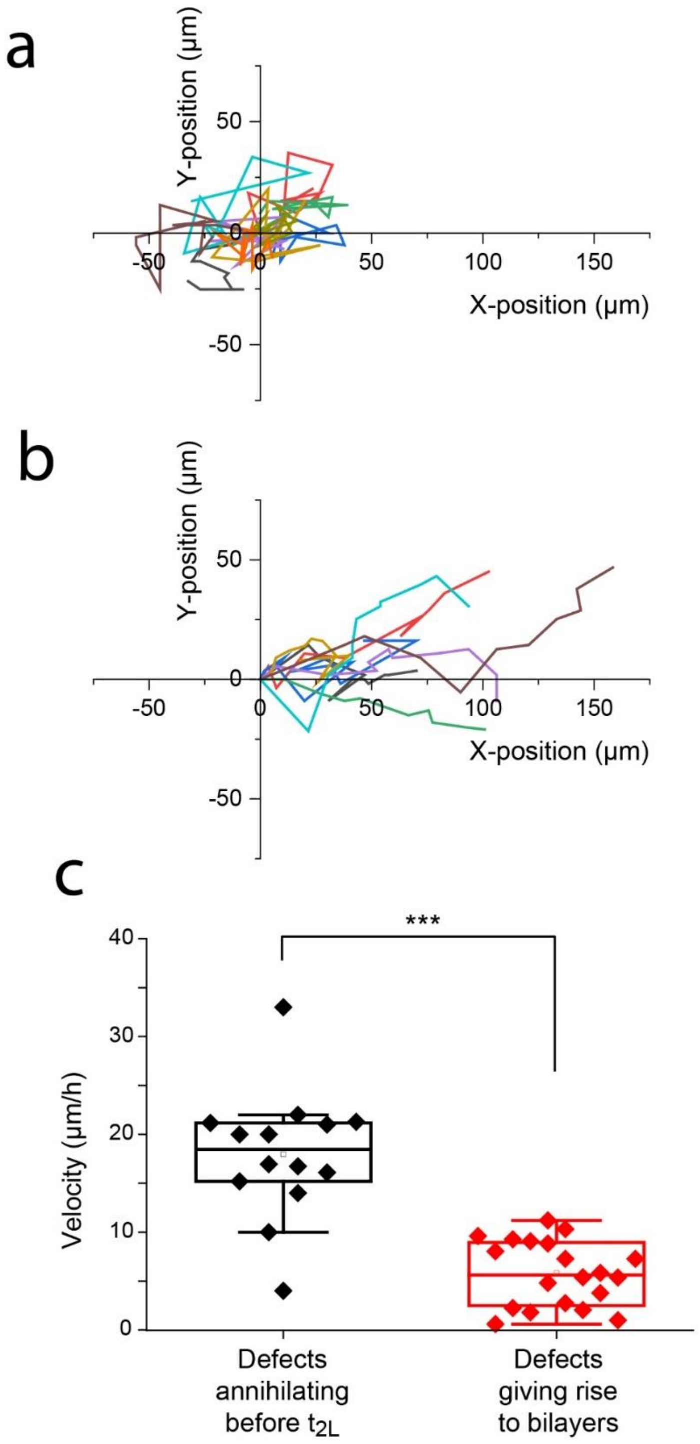


**<u>Figure S4:</u>** Representative trajectories of +1/2 defects giving rise to bilayers (a) or annihilating before $t_{2L}$ (b) over a time course of 5 h. Defects that eventually annihilate before reaching $t_{2L}$ behave as self-propelled particles while those that survive until $t_{2L}$ and give rise to crisscross bilayer remain in place. c) Comparison of the velocities of the defects that give rise to multilayers at $t_{2L}$ (red points) with the ones that annihilate before $t_{2L}$ (black points). In both cases, the speed here is defined as the ratio of the end-to-end distance to the corresponding time. Here, defects giving rise to bilayering were monitored between their creation and the onset of bilayering; defects that annihilated before $t_{2L}$ were monitored from their creation to their annihilation.

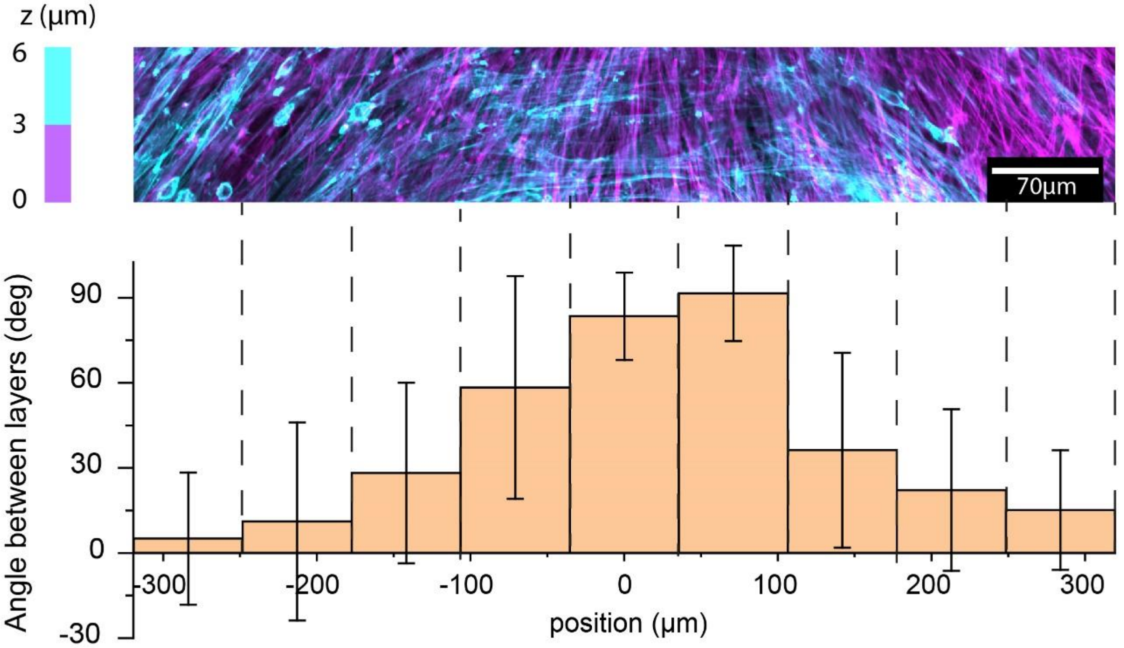


**Figure S5**: Relative angle profile between layer 2 and layer 1 at $t_{2L}$+10h. Confocal actin image of the two superimposed layers and corresponding profile of their relative angles. The angle between layers is defined as sgn(x)*[angle(layer2)-angle(layer1)].

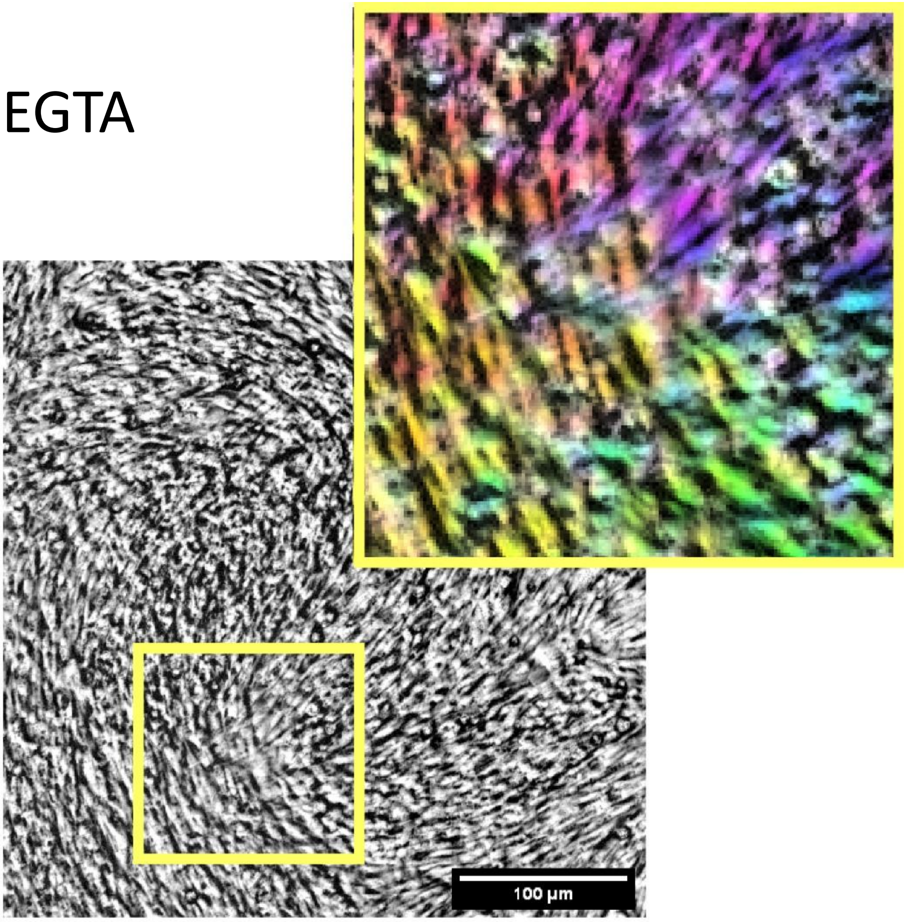


**Figure S6:** Crisscross bilayer in presence of EGTA (live cells, phase contrast, 20 hours post confluency). EGTA (2.5 mM) was introduced in the medium after cells have reached confluence. Although it impairs cadherin-mediated cell-cell adhesions, EGTA does not abrogate the crisscross structure (see inset where the orientation is color-coded, and the typical orthogonal structure is visible) (3 replicates).

RPE1 cells

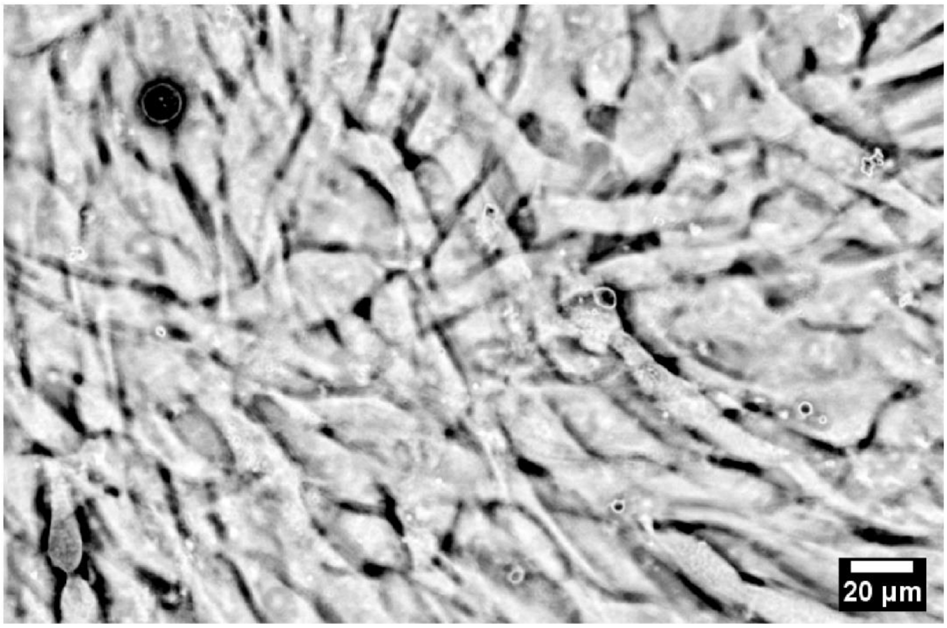


**Figure S7**: Retinal Pigment Epithelial (RPE1) cells crisscross similarly to C2C12 cells. Live cells, phase contrast image. $t = t_{2L} + 36h$

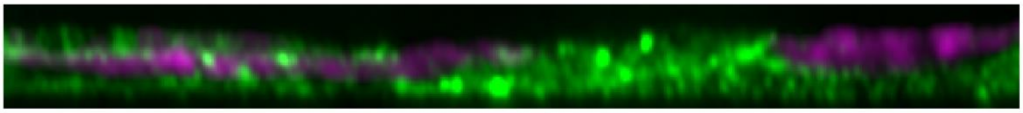


**Figure S8**: Secretion of collagen (green) by C2C12 cells. x-z view. Fixed cells t = $t_{1L}$+20h. Nuclei are in magenta.

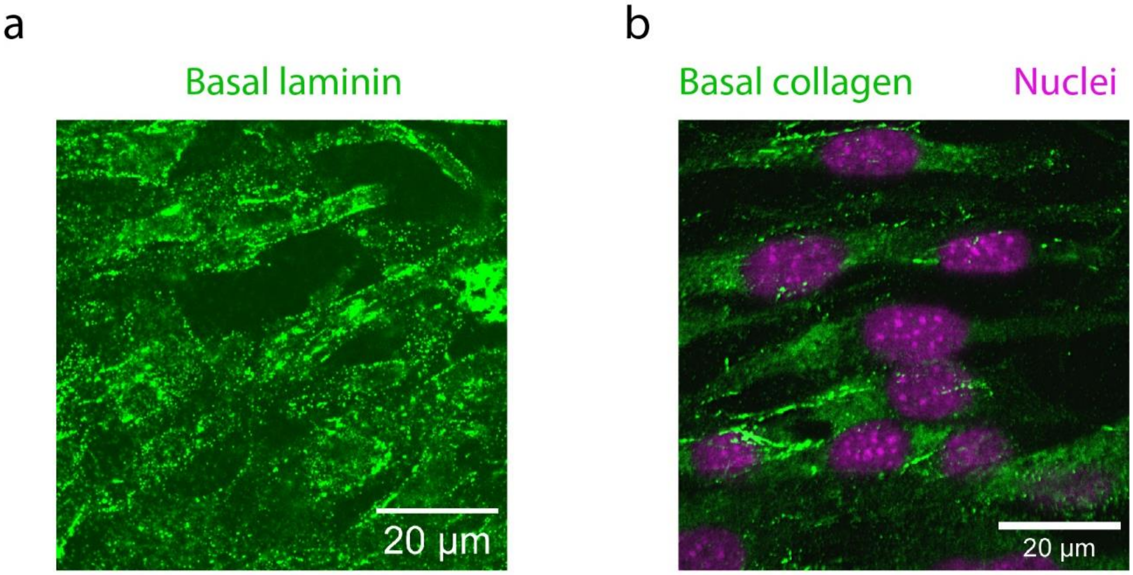


**<u>Figure S9</u>** a,b/ Secreted laminin (a) and collagen (b) at the basal side of layer 1 (on the glass surface). Fixed cells. Monolayer ($t \sim t_{2L}$). Note the orientation of the ecm fibers that matches the one of the cells in the monolayer. These oriented fibrils coexist with a more punctuated distribution. Fixed cells; A/ laminin green, b/collagen green, nuclei magenta.

| Target | Type | Reference | Dilution |
|---|---|---|---|
| Laminin α1 and α2 chains | Rabbit Laminin poly 1+2 | ab7463(abcam) | 1:100 |
| Paxillin | Rabbit mono(with Alexa Fluor®647) | ab246719(abcam) | 1:50 |
| Collagen IV | Rabbit polyclonal to Collagen IV | ab6586(abcam) | 1:100 |
| N-cadherin | Mouse Monoclonal | 33-3900(Thermofisher) | 1:50 |
| Actin | Phalloidin-TRITC | P1951 (Sigma-Aldrich) | 10 μM |
| Actin | Sir-actin | SC001 (Spirochrome) | 0.1 μM |
| Fibronectin | Rabbit polyclonal to fibronectin | F3648 (Sigma) | 1:100 |
| Nucleus | Sir-DNA | SC007 (Spirochrome) | 200 nM |

| Name | Type | Dilution |
|---|---|---|
| Mouse Secondary Antibody | GoatXMouse Alexa Fluor PLUS 647(A32728) (Thermofisher) | 1:200-1:300 |
| Rabbit Secondary Antibody | ChickenXRabbit Alexa Fluor 488(A-21441) (Thermofisher) | 1:100 |

**Table S1**

Reactants and concentrations used in the present work.

**Movie S1:**

Before the bilayering and during this process, complex flows can be visualized on the sides of the comet on top of the global progression of the cells forming layer 2. For this movie, 10% of the cells were Actin-mCherry so that they could be tracked independently. 15 min between successive frames. See an analysis of the trajectories in Figure 4f.

**Movie S2 :**

Progression of the bilayering process with time. Phase contrast movie. Note the crisscross orientation of the bilayer. See corresponding sequence in Figure 3.

**Movie S3**

Progression of the bilayering process with time. Confocal movie on live SiR-Actin stained cells. The cells keep their orientation during the bilayering which results in the crisscross orientation of the bilayer. The magenta color codes for heights just above the glass surface (h=1.4 ± 1.4 µm). The cyan color codes for heights of 8.4 ± 1.4 µm above the glass surface (within layer 2). The defect outline in the first images is a guide for the eye.

**Movie S4**

Addition of collagenase impairs bilayering and destroys the partial bilayers that have started to form. Collagenase was added at the onset of bilayering (just before the start of this video). 15 min between successive frames. 3 replicates
